# Diagnostic Value of Urinary Microalbumin Level in Postpartum Acute Kidney Injury

**Published:** 2018-03

**Authors:** Jian ZHANG, Ning LI, Wenling GUO, Chuanyan MA, Guangcheng ZHAO

**Affiliations:** 1. Dept. of Obstetrical, Binzhou Central Hospital, Binzhou, Shandong, China; 2. Dept. of Imaging, Binzhou Central Hospital, Binzhou, Shandong, China

**Keywords:** Postpartum acute kidney injury, Urinary microalbumin, Diagnosis

## Abstract

**Background:**

We aimed to explore the diagnostic value of urinary microalbumin (mALB) level in postpartum acute kidney injury.

**Methods:**

A total of 127 maternity patients were selected from December 2013 to January in 2016 in Binzhou Central Hospital, Binzhou, Shandong, China and divided into two groups: the kidney injury and normal kidney group. The dynamic changes and diagnostic value of urine microprotein in postpartum acute kidney injury were analyzed.

**Results:**

The postpartum mean arterial pressure of maternity patients in the kidney injury group was 104.3 ± 11.6 mmHg, which was significantly higher than that of the normal kidney group (*P*<0.05). The mean age of the kidney injury group was 32.3 ± 11.6 years, which was significantly higher than that of the normal kidney group (*P*=0.006). In the kidney injury group, the postpartum glomerular filtration rate (GFR) was 78.4 ± 11.5 mL/min, which was significantly lower than the normal group (*P*=0.001), and urinary microalbumin was 2.87 ± 1.24 mg/mmol·Cr. The difference was statistically significant (*P*=0.002). mALB/GFR, Cr, urinary mALB, and GFR were the independent risk factors of postpartum acute kidney injury. The area under the ROC curve for mALB/GFR was 0.759, whereas the area under the ROC curve for Cr was 0.681, which was smaller (*P* = 0.042). The area under the ROC curve of mALB was 0.785 (*P*=0.027), which was close to the area under the ROC curve of mALB/GFR.

**Conclusion:**

Urinary mALB test is noninvasive and has high diagnostic value for postpartum kidney injury.

## Introduction

Postpartum acute kidney injury is a clinical syndrome denoted by decreased glomerular filtration rate (GFR), increased proteinuria, and renal dys-function from a variety of causes. However, patients do not present with apparent clinical manifestations of renal dysfunction during pregnancy or before giving birth (1). Unlike eclampsia, postpartum kidney injury occurs later, and is more occult. Obstetricians often pay less attention to this condition. Therefore, it is often overlooked in clinic (2, 3). However, it may cause acute renal failure within a short time after delivery, seriously threatening the health and lives of patients. Renal function injury can occur in other situations where renal perfusion decreases because of postpartum hemorrhage. This form of renal function injury can usually be reversed by supplementing blood volume (4). In recent years, with increased requirements for improved maternal perinatal quality of life, obstetricians pay more attention to postpartum acute kidney injury (5).

According to our clinical observations, postpartum blood urea nitrogen (BUN) and creatinine (Cr) were high to some extent in maternity patients without postpartum kidney injury, which might be associated with birth injury or muscle contraction. The false positive results derived from the aforementioned situations can be distracting for early diagnosis of postpartum acute kidney injury. Urinary microalbumin (mALB) is a noninvasive diagnostic marker that has previously been shown to have good sensitivity and specificity (6) for the diagnosis of kidney injury caused by neonatal hypoxic-ischemic encephalopathy.

The aim of the present study was to investigate the clinical value of urinary mALB in the diagnosis of postpartum acute kidney injury.

## Methods

A total of 127 maternity patients with normal labor in the Obstetrical Department of Binzhou Central Hospital, Binzhou, Shandong, China were selected from December 2013 to January 2016. Among the 127 maternity patients, 31 experienced elevated kidney function that lasted 48 h without apparent improvement, and were diagnosed with postpartum acute kidney injury. The maternity patients were divided into two groups: the kidney injury group and normal kidney group. Informed consent was obtained from the patients and their families. The inclusion criteria were as follows: 1. over 22 years old; 2. full term, normal labor; 3. diagnosis of acute kidney injury. The exclusion criteria were as follows: 1. prenatal kidney injury or suspected kidney injury; 2. preterm labor or induced labor; 3. test results were severely affected by postpartum lochia contamination.

The study was approved by the Ethics Committee of Binzhou Central Hospital and written informed consents were signed by the patients and/or guardians.

The diagnostic criteria for acute kidney injury were as follows: 1. a clinical syndrome characterized by sudden (1–7 d) and sustained (>24 h) reduction in renal function; 2. An increase of serum creatinine (SCr) by over 0.5 mg/dL; 3. Showing clinical symptoms of azotemia, and disturbances of water-electrolyte and acid-base balance; 4. May present with oliguria (<400 mL/24 h or 17 mL/h) or anuria (<100 mL/24 h).

Midstream fasting urine samples (10 mL) were collected in the morning from maternity patients, 24 h before and 48 h after labor. Urine samples were centrifuged at 1,500 rpm for 10 min, and the supernatant was collected to test for physiological parameters such as urinary mALB, Cr level, GFR, urine glucose (GLU), and white blood cell count (WBC). Among these tests, urinary mALB was determined with a rate nephelometric immunoassay using a Beckman Array Protein System 360. The measurement was carried out according to the manufacturer’s instructions and the urine mALB kit (Beijing Zhongshan Golden Bridge Biotechnology Co., Ltd., Beijing, China). According to the reference range provided by the manual, an albumin level of higher than 19.0 mg/L was regarded as positive. For blood tests, peripheral blood samples were collected from maternity patients 24 h before and 48 h after labor. Blood samples were centrifuged at 4°C, and the supernatant was collected for measuring BUN. Except for mALB, the other urine and blood tests were performed using an Abbott Architect C16000 clinical chemistry analyzer. Mean arterial pressure (MAP) was measured using a YUWELL YE680A electronic sphygmomanometer.

### Statistical analysis

Statistical analysis was performed using the SPSS 13.0 (SPSS Inc., IL, USA) software package. Experimental data are presented as mean ± standard deviation. Independent samples χ^2^-test was used for comparisons between groups. *P*<0.05 was considered statistically significant. Pearson’s correlation coefficient was used for correlation analyses. Multivariate analysis was performed using the logistic regression model. The diagnostic value of the urinary mALB test in postpartum acute kidney injury was evaluated by log-rank test.

## Results

### General comparison of maternity patients in the two groups

There were no significant differences in the numbers of pregnancies and deliveries between the two groups (*P*>0.05). The mean age of the kidney injury group was significantly higher than that of the normal kidney group (*P*<0.05), (Table 1).

**Table 1:** General comparison of maternity patients in the two groups

***Group***	***Cases***	***Mean Age (yr)***	***Number of Pregnancies***	***Number of Deliveries***
Kidney Injury	31	32.31 ± 11.63	3.22 ± 0.68	2.11 ± 0.54
Kidney Normal	96	25.75 ± 4.82	3.82 ± 0.73	2.23 ± 1.24
χ^2^ Value		8.5	0.33	0.22
*P*-value		0.006	0.682	0.719

### Comparison of various physiological parameters before and after labor in the two groups

There were no significant differences in physiological parameters between the two groups before labor. The postpartum levels of MAP, Cr, BUN, GFR, and mALB in the kidney injury group were significantly higher than the corresponding prenatal levels in the same group and the postpartum levels in the normal kidney group (*P*<0.001). There were no significant differences in the levels of GLU and WBC before and after labor in the two groups (Table 2).

**Table 2:** Comparison of various physiological parameters before and after labor in the two groups

***Physiological Parameter***	***Kidney Injury Group (n = 31)***		***Normal Kidney Group (n = 96)***	

	Before Labor	After Labor	Before Labor	After Labor
MAP (mmHg)	81.12±12.21	104.34±11.62*#	83.32±12.81	81.34±10.91
Cr (μmol/L)	93.21±12.23	107.89 ± 26.17*#	94.32±10.93	97.60 ± 16.57
BUN (mmol/L)	5.32±1.21	6.43±2.14*#	5.12±0.48	5.20 ± 1.56
GFR (mL/min/1.73 m^2^)	93.31±21.92	65.54±16.83*#	93.4±11.93	95.58 ± 21.16
mALB (mg/g)	28.32±10.21	51.78±12.02 *#	29.31±18.33	29.72±14.74
GLU (mmol/L)	5.37±2.1	5.26±1.62	5.21±1.23	5.79±2.05
WBC (×10^9^)	7.92±1.3	7.28±1.29	8.32±1.23	8.03 ± 5.71

* *P*<0.05 compared with parameters before labor;

# *P*<0.05 compared with parameters of the normal kidney group after labor

### Comparison of postpartum glomerular filtration rate and urinary microalbumin level in the two groups

The postpartum GFR and urinary mALB level were compared between the kidney injury group and normal kidney group.

As shown in Table 3, the postpartum GFR in the kidney injury group was significantly lower than in the normal kidney group, whereas the postpartum urinary mALB level was significantly higher in the corresponding comparison. The differences were statistically significant (*P*<0.001).

**Table 3:** Comparison of postpartum glomerular filtration rate and urinary microalbumin level in the two groups

***Group***	***Cases***	***GFR (mL/min)***	***Urinary Microalbumin (mg/L)***	***Urinary Microalbumin/GFR***
Normal Kidney	96	124.53±2.72	7.25±2.33	0.08±0.13
Kidney Injury	31	78.42±11.56	24.32±7.81	0.25±0.14
*t*-value		8.41	9.22	7.63
*P*-value		0.001	0.002	0.004

### Multivariate logistic regression analysis

Unconditional multivariate logistic regression analysis was carried out, in which the independent variables were those shown by univariate analysis to be statistically significant, such as MAP, mALB/GFR, Cr, mALB, BUN, and GFR. The dependent variable was the occurrence of postpartum kidney injury (y=0, 1). As shown in Table 4, mALB/GFR, Cr, and mALB were the independent risk factors of postpartum acute kidney injury.

**Table 4:** Multivariate logistic regression analysis

***Risk Factors***	***β***	***SE***	***Wald Value***	***P-value***	***OR Value***	***95% CI***
mALB/GFR	1.86	0.48	17.5	0.003	7.19	2.73–17.76
Cr	0.19	0.22	2.11	0.017	1.34	0.89–2.01
mALB	0.19	0.07	2.23	0.027	1.09	0.97–1.22
BUN	0.08	0.01	3.57	0.059	1.05	0.97–1.09
MAP	1.29	0.36	0.11	0.371	1.02	0.33–1.21
GFR	0.37	0.42	0.08	0.736	1.26	0.97–1.48

### ROC curve

**Fig. 1:**
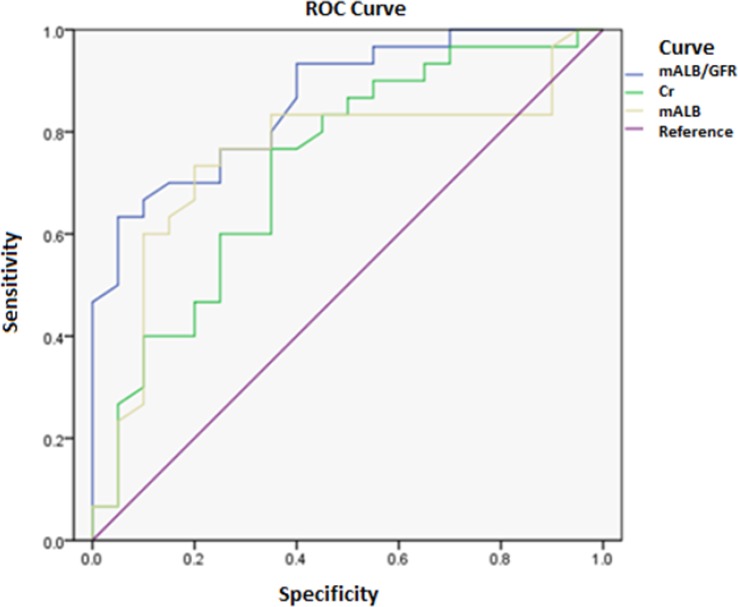
ROC curves of mALB/GFR, Cr, and mALB

An ROC curve was plotted to analyze the diagnostic value of the commonly used parameters of renal function (Cr, mALB/GFR, and mALB) for postpartum acute kidney injury (Fig. 1).

The area under the ROC curve for mALB/GFR was 0.759 (*P*<0.001), whereas the area under the ROC curve for Cr was 0.681, which was smaller (Z value = 2.077, 95% CI = 0.008–0.328, *P*= 0.042). The area under the ROC curve of mALB was 0.785 (*P*<0.05), which was close to the area under the ROC curve of mALB/GFR (*P*<0.01).

## Discussion

At present, the cause of postpartum acute kidney injury remains unclear. Based on published studies6, postpartum hemorrhage, decreased perfusion, inflammatory factors from a variety of stress responses during labor, and glomerular deposition of immunoglobulins may cause kidney injury, leading to damage of glomerular epithelial cells and basement membranes. Acute kidney injury for maternity patients is sudden in onset and clinically occult. Patients may experience unexplained edema and oliguria after giving birth, which seriously affects the quality of life of patients. Therefore, early diagnosis of postpartum acute kidney injury is important (7–9).

Urinary microproteins mainly include trace amounts of albumin, transferrin (TRF), IgG, and α-1-microglobulin. Microalbuminuria refers to the presence of microalbumin in urine (10). Albumin is a protein in blood, and under normal physiological conditions, only a very small amount of albumin is found in urine. Tests for microalbuminuria can detect abnormal renal leakage of protein (11). In diabetic nephropathy and hypertensive nephropathy, microalbumin level is used as an early biomarker of kidney damage (12–14), although its application in post-partum acute kidney injury is still limited.

In this study, the mean age of the 31 maternity patients with postpartum acute kidney injury was significantly higher than that of the normal kidney group, suggesting that advanced maternal age may increase the incidence of postpartum kidney injury. The postpartum MAP of the kidney injury group was also markedly higher than that of the normal kidney group. MAP elevation may be associated with bleeding, sympathetic nervous system excitability, and other factors, which may affect renal perfusion, causing postpartum acute kidney injury. These results were consistent with published findings. In pregnant women with kidney injury, the urinary mALB and TRF levels in mid-pregnancy (13–28 weeks of pregnancy) were significantly higher (*P*<0.001) than those of healthy non-pregnant women as measured by rate nephelometry (10). Moreover, other parameters were comparable. However, the levels of all four urinary microproteins of late pregnancy patients (28–40 weeks of pregnancy) and pregnancy-induced hypertension patients (20–38 yr old) were markedly elevated. In addition, the urinary mALB/GFR ratio was highly useful for the diagnosis of contrast-induced nephropathy (11). When the ratio was 1.17, the diagnostic sensitivity and specificity of contrast-induced nephropathy were 94.1% and 72.5%, respectively. This clearly indicated that the urinary mALB/GFR ratio represents a useful biomarker for the diagnosis of contrast-induced nephropathy.

In this study, the area under the ROC curve for mALB GFR was 0.759, whereas the area under the ROC curve for Cr was 0.681, which was smaller. The area under the ROC curve of mALB was 0.785, which was close to the area under the ROC curve of mALB/GFR. These results demonstrated that mALB has high sensitivity and specificity for the diagnosis of postpartum acute kidney injury and is of high clinical value.

Urinary microalbumin is a sensitive biomarker of renal injury that is of great significance for early diagnosis of renal injury in patients with preeclampsia. It is of clinical significance to diagnose acute kidney injury by post-delivery laboratory tests of Cr and BUN (6, 7). However, during labor, BUN and Cr levels may be affected by consistent contraction of skeletal muscle, whereas the increase of urinary mALB level is mostly because of glomerular filtration dysfunction in kidney injury. Therefore, compared with the commonly used Cr and BUN, urinary microprotein tests can effectively improve diagnosis of post-partum acute kidney injury. However, it was noted that during labor, degradation of skeletal muscle proteins and smooth muscle proteins can affect postpartum microglobulin excretion, whereas they have limited effect on BUN and Cr levels. The false positive rate is relatively low. Thus, it is believed that urinary microproteins could be used as physiological parameters for the diagnosis of postpartum acute kidney injury.

## Conclusion

Urinary mALB can be used as a valuable biomarker in the non-invasive diagnosis of postpartum acute kidney injury. However, its underlining mechanism and sensitivity still require further studies.

## Ethical considerations

Ethical issues (Including plagiarism, informed consent, misconduct, data fabrication and/or falsification, double publication and/or submission, redundancy, etc.) have been completely observed by the authors.
